# Burkitt Lymphoma Presenting as Unilateral Deafness in an Immunocompetent Patient

**DOI:** 10.1155/2012/369264

**Published:** 2012-09-25

**Authors:** Andre Pinto, Offiong Francis Ikpatt, Jennifer Chapman-Fredericks

**Affiliations:** Department of Pathology, Jackson Health System, University of Miami, Miami, FL 33131, USA

## Abstract

A 55-year-old HIV-negative white male presented with right ear deafness, right axillary lymphadenopathy, and weight loss. Laboratory findings included anemia, marked leukocytosis, and thrombocytopenia. Examination of the peripheral smear demonstrated the presence of increased circulating blast-like cells of intermediate size, with basophilic cytoplasm and nuclei with open chromatin. MRI of the brain was compatible with hemorrhagic labyrinthitis. Excisional biopsy of the axillary mass revealed an enlarged lymph node with effaced architecture and “starry sky” appearance. The cells expressed CD20, CD10, BCL6, and surface kappa immunoglobulin light chain, with a high proliferative index by immunohistochemistry and flow cytometry. Subsequent bone marrow biopsy was hypercellular (approximately 95%), with blast-like cells virtually replacing all hematopoietic elements. Routine karyotype as well as FISH analysis of bone marrow cells demonstrated rearrangement of the MYC gene at chromosome 8q24 region, IGH/MYC fusion, and additional signal for IGH gene. We present herein a case of sporadic Burkitt lymphoma occurring in a previously healthy HIV-negative male. The unusual clinical findings in this case include the relatively older age at presentation (55 years), an immunocompetent patient who had nodal involvement and leukemic phase of Burkitt, coupled with partial deafness. A brief educational review of this neoplasm is made.

## 1. Introduction

Non-Hodgkin lymphomas (NHLs) can be divided into two prognostic groups: the indolent lymphomas and the aggressive lymphomas. Indolent NHL types have a relatively good prognosis, with a median survival as long as 10 years, but they usually are not curable in advanced clinical stages. The aggressive types of NHL have a more rapidly progressive clinical course, but a significant number of these patients can be cured with intensive combination chemotherapy regimens. In general, patients with aggressive NHL have roughly 30% to 60% cure rates with modern therapies [1] with the vast majority of relapses occurring within the first 2 years after initiation of therapy [2]. 

Patients who present with or convert to aggressive forms of NHL may have sustained complete remissions with combination chemotherapy regimens or aggressive consolidation with marrow or stem cell support [3, 4]. Lymphomas with aggressive biologic behavior include diffuse large B cell lymphoma (DLBCL), Burkitt lymphoma (BL), T-cell lymphomas, and precursor B and T acute lymphoblastic leukemia/lymphoma.

This is a case presentation of a 55-year-old white male who developed progressive unilateral hearing loss and axillary lymphadenopathy. The patient was subsequently diagnosed with nodal Burkitt lymphoma, sporadic type, and was found to have bone marrow involvement at the time of initial staging. 

In the postgenomic era, the diagnosis of BL requires the integration of key morphologic findings with immunophenotypic properties, proliferation assessment, and demonstration of the presence of certain molecular findings and lack of others. The purpose of this paper is to review a case of sporadic BL that initially presented with an unusual otologic manifestation as well as to review the recent literature concerning the diagnosis and biology of BL. 

## 2. Material and Methods

**Figure 1:**
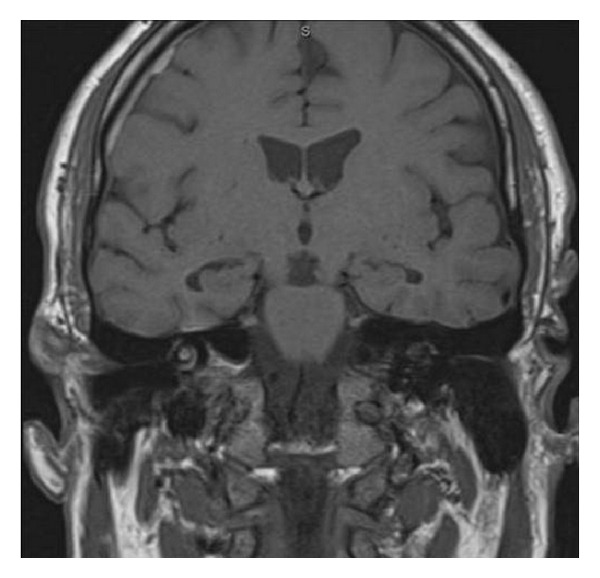
Brain MRI showing hypersignal in the inner ear compatible with hemorrhagic labyrinthitis.

We present herein a case of a 55-year-old HIV-negative white male with no significant past medical history, who initially developed right ear deafness and tinnitus of a relatively abrupt onset (less than one week). He denied pain, fever, or any other symptoms. Upon review of systems, he also had right axillary lymphadenopathy and complained of weight loss. On physical examination, no lymph node enlargement was obvious and his liver and spleen were not increased in size. CT scans of the abdomen, chest, and pelvis were negative. MRI of the brain showed abnormal high signal in the cochlea, vestibule, and semicircular canals compatible with hemorrhagic labyrinthitis (Figure 1) Multiple CSF analyses were negative for infection or malignant cells. Laboratory findings included a mild anemia (Hgb 9.3 g/dL), elevated WBC of 47.5 × 10³/*μ*L, and thrombocytopenia (PLT 41 × 10^3^/*μ*L). Elevated levels of LDH (3227 units/L) and uric acid (12.6 mg/dL) were noted. 

Patient samples collected and reviewed included peripheral blood, bone marrow aspirate, and bone marrow biopsy. Comprehensive analysis of these samples including histologic examination, immunohistochemistry, flow cytometric analysis, and molecular genetic assays was performed at and interpreted by physicians at the University of Miami.

## 3. Results

**Figure 2:**
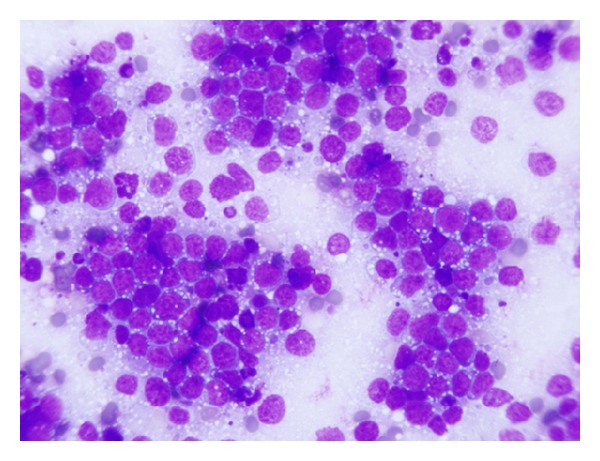
Peripheral smear, Wright-Giemsa, 200x. Absolute lymphocytosis due to increased monotonous, medium-to-large, lymphoid-appearing cells with high nuclear-to-cytoplasmic ratios, immature-appearing chromatin pattern with visible nucleoli.

Examination of the peripheral smear (Figure 2) revealed an absolute lymphocytosis due to the presence of increased circulating blast-like cells of intermediate size, with vacuolated, basophilic cytoplasm, and immature-appearing nuclei with open chromatin (77%).

**Figure 3:**
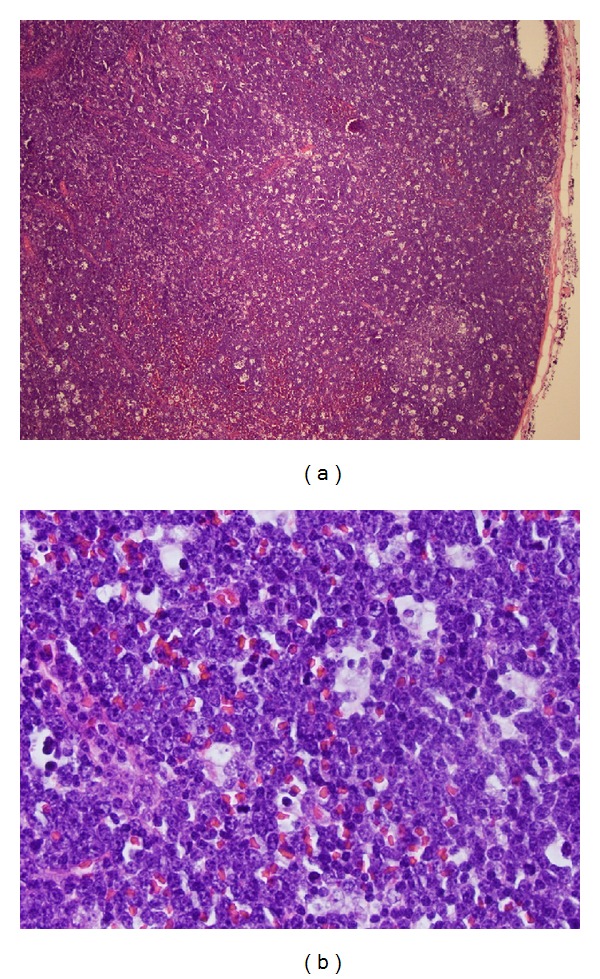
(a) Lymph node biopsy, H&E, 40x. Effaced nodal architecture due to large tumor mass which has a “starry sky” appearance on low power microscopy due to abundant tingible body macrophages, which are a reflection of the rapid proliferation and cell turnover associated with this tumor. (b) Lymph node biopsy, H&E, 400x. Monotonous population of immature-appearing lymphoid cells with vesicular chromatin and multiple nucleoli. Cells are closely approximated due to lack of abundant cytoplasm. Several tingible body macrophages are present as are several mitotic figures.

An excisional biopsy of the axillary lymph node showed an enlarged lymph node with effaced nodal architecture. A “starry sky” appearance (Figure 3(a)) was apparent on low power magnification. Oil immersion microscopy identified a monotonous population of tumor cells of intermediate cell size diffusely replacing the nodal tissue. Cells were closely approximated due to lack of significant cytoplasm and had round to oval nuclei with open chromatin and multiple nucleoli (Figure 3(b)). These cells expressed CD20, CD10, BCL6, and surface kappa immunoglobulin light chain, with a high proliferative index (high Ki67 expression of >90%) by immunohistochemistry and flow cytometry. They were negative for CD34, TdT, BCL2, CD3, CD5, and other tested markers. 

**Figure 4:**
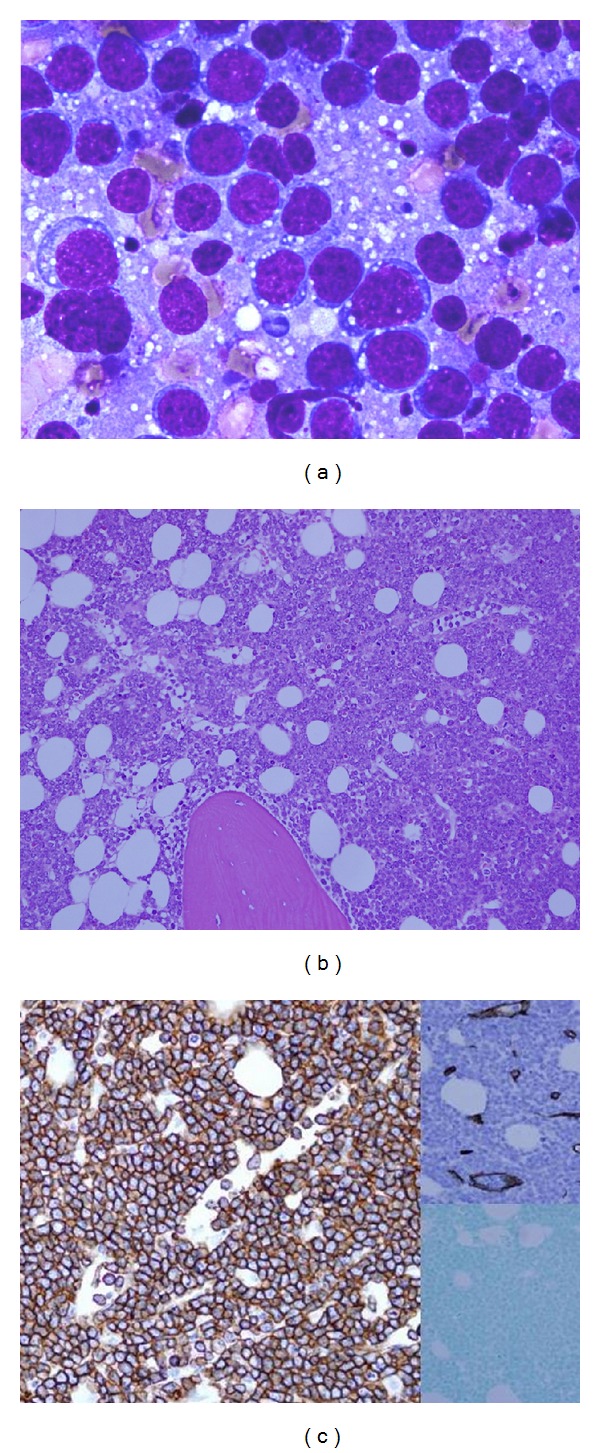
(a): Bone marrow aspirate, Wright Gimsea, 400x. Monotonous population of medium-to-large-sized lymphoid cells with high nuclear-to-cytoplasmic ratio and immature appearing chromatin with multiple nucleoli. Basophilic cytoplasm with multiple cytoplasmic vacuoles is apparent. (b) and (c) Bone marrow core biopsy, H&E, 200x. Hypercellular bone marrow due to a neoplastic cell infiltrate. The cells are present in a diffuse pattern and are positive for CD20 (left) and negative for blast markers including CD34 (upper right) and TdT (lower right) by immunohistochemistry.

Subsequent bone marrow aspiration (Figure 4(a)) showed a prominent population of intermediate-sized blast-like cells with a high nuclear-to-cytoplasmic ratio, vesicular chromatin pattern, prominent nucleoli, and a PAS positive vacuolated cytoplasm. The bone marrow core biopsy (Figure 4(b)) was hypercellular (cellularity of approximately 95%), consisting of blast-like cells similar to those observed in the aspirate, virtually replacing all normal hematopoietic cells.

**Figure 5:**
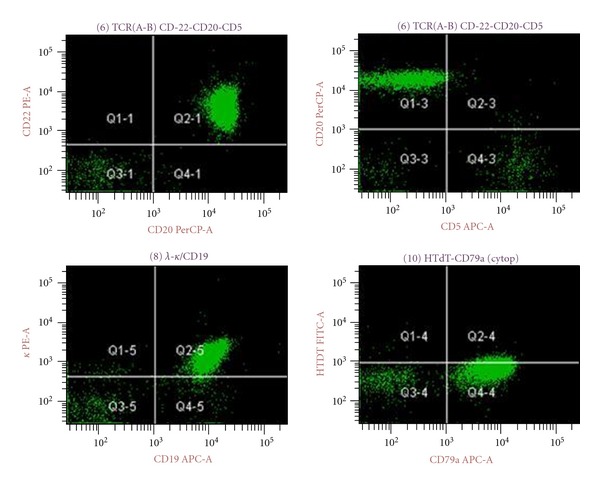
Flow cytometry scatter plots. The malignant cell population is positive for CD20, CD22, CD19, and CD79a and demonstrated kappa surface light chain restriction. The cells are negative for CD5 and TdT.

Flow cytometric analysis of the bone marrow aspirate revealed a predominant population of mature B lymphocytes that exhibited kappa-restricted surface immunoglobulin light chain expression and expressed CD79a, CD19, CD20, CD22, HLADR, and CD10. There was no expression of either CD34 or TdT (Figures 4(c) and 5).

**Figure 6:**
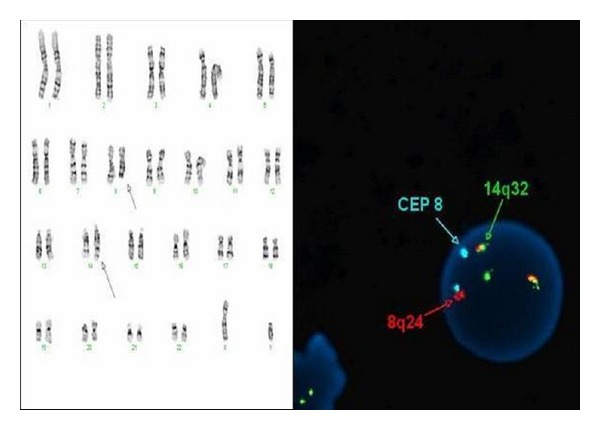
Karyotype (left) with arrows pointing translocation (8, 14), highlighted by FISH (dual fusion probe) study (right): the IGH gene (14q32) is in green, the MYC gene (8q24) is red, and the centromere 8 (CEP 8) is in aqua. If the patient is normal, the expected pattern would be 2 reds, 2 greens, and 2 aqua signals. The abnormal pattern is two fusions (red and green together), 1 red (normal 8), 1 green (normal 14), and 2 aqua (the two centromeres of chromosome 8).

Routine karyotype as well as FISH analysis of bone marrow cells demonstrated rearrangement of the MYC gene at chromosome 8q24 region (33.5% of cells examined), IGH/MYC fusion (33% of cells examined), and additional signal for IGH gene (28.5% of cells examined). Signals for IGH/BCL2 fusion were not detected (Figure 6).

Based on the above morphologic findings, immunophenotype, and on the presence of a MYC translocation in the absence of an IGH/BCL2 translocation and other cytogenetic findings, the diagnosis of BL was made. The patient was started on chemotherapy of hyper-CVAD plus Rituximab regimen, with five prophylactic doses of intrathecal methotrexate and cytarabine. After two months of treatment and three sessions of chemotherapy, the symptoms of unilateral deafness still persisted. The patient subsequently lost followup in our service. 

## 4. Discussion

The World Health Organization Classification of Lymphoid Neoplasms identifies BL as a highly aggressive mature B-cell neoplasm [5]. The lymphoma and the leukemic forms are described as different manifestations of the same disease, and the diagnostic criteria have evolved with time and now require incorporation of morphologic, immunophenotypic, cytogenetic, and molecular diagnostic techniques, as demonstrated in the above-presented case [6]. 

First described by Dennis Burkitt in 1958, BL is a highly aggressive non-Hodgkin lymphoma (NHL) often presenting in extranodal sites [7]. Three clinical variants of BL are described: endemic, sporadic, and immunodeficiency-associated types. The endemic form of BL classically affects children in equatorial Africa and is causally associated with Epstein-Barr virus (EBV) infection. Patients typically present with involvement of the jaw, kidneys, gastrointestinal tract, ovaries, breast, or other extranodal sites [8, 9]. Sporadic BL occurs worldwide; it represents less than 2% of lymphoma in adults and up to 40% of lymphoma in children in the USA and western Europe [10]. The abdomen, especially the ileocecal area, is the most common site of involvement; the ovaries, kidneys, omentum, Waldeyer's ring, and other sites may also be involved [10]. The immunodeficiency subtype is frequently observed in the setting of human immunodeficiency virus (HIV) infection and, unlike other HIV-related lymphomas, is frequently noted in patients with CD4 counts exceeding 200 cells/*μ*L [11]. Although secondary involvement of bone marrow and peripheral blood by a typical BL may occur in some patients in advanced stages of bulky disease, an exclusive, pure leukemic presentation with primary bone marrow disease (pure Burkitt leukemia) is very unconventional [12]. 

Despite their clinical diversity, striking biologic similarities exist between the defined variants of BL (sporadic, endemic, and immunodeficiency related). Indeed, all variants of BL share the same morphologic and immunophenotypic findings. Additionally, almost all variants typically demonstrate MYC translocations which are most commonly balanced translocations involving the MYC locus at chromosome 8q24 and either the immunoglobulin heavy chain gene at chromosome 14q32 or one of the two immunoglobulin light chain genes at chromosome 2p12 (kappa) or 22q11 (lambda). Of these, the translocation (8; 14) (q24; q32) is by far the most common translocation seen in all clinical subtypes of BL, occurring in 80–90% of cases [13]. 

Regarding the association between leukemia and hearing loss, this is not a novel observation. There have been previous clinical and histopathologic reports concerning the occurrence hearing loss in leukemia, including conductive and sensorial hearing loss, facial palsy, and ear infections [14]. These may be the result of leukemic infiltration, hemorrhage, and/or inflammatory changes [15]. A recent study of acute lymphoblastic leukemia (ALL) found that 69% of patients had definite otologic complaints during the course of the disease. Hemorrhage was observed most commonly in the middle ear in the submucous membranes, also hypervascularization and capillary dilatation. Extensive hemorrhage in the cochlea and vestibule was observed in 2 out of 13 patients [16]. To our knowledge, however, there are no previous reports of BL presenting with unilateral deafness.

In summary, BL is an aggressive form of lymphoma that can rarely affect older immunocompetent patients and should be part of the differential diagnosis of both solid and leukemic phases of hematologic malignancies. Finally, involvement by a neoplastic process should be considered in the differential diagnosis of patients presenting with hemorrhagic labyrinthitis and/or acute unilateral hearing loss.
